# Transcriptome Profiling and Expression Localization of Key Sex-Related Genes in a Socially-Controlled Hermaphroditic Clownfish, *Amphiprion clarkii*

**DOI:** 10.3390/ijms23169085

**Published:** 2022-08-13

**Authors:** Huan Wang, Meng Qu, Wei Tang, Shufang Liu, Shaoxiong Ding

**Affiliations:** 1 Yellow Sea Fisheries Research Institute, Chinese Academy of Fishery Sciences, Qingdao 266071, China; 2 Xiamen Key Laboratory of Urban Sea Ecological Conservation and Restoration, College of Ocean and Earth Sciences, Xiamen University, Xiamen 361005, China; 3 CAS Key Laboratory of Tropical Marine Bio-Resources and Ecology, South China Sea Institute of Oceanology, Chinese Academy of Sciences, Southern Marine Science and Engineering Guangdong Laboratory (GML, Guangzhou), Guangzhou 511458, China

**Keywords:** protandry, socially-controlled sexual development, clownfish, transcriptome, expression localization, *dmrt1* gene

## Abstract

Clownfish can be an excellent research model for investigating the socially-controlled sexual development of sequential hermaphrodite teleosts. However, the molecular cascades underlying the social cues that orchestrate the sexual development process remain poorly understood. Here, we performed a comparative transcriptomic analysis of gonads from females, males, and nonbreeders of *Amphiprion clarkii*, which constitute a complete social group, allowing us to investigate the molecular regulatory network under social control. Our analysis highlighted that the gonads of nonbreeders and males exhibited high similarities but were far from females, both in global transcriptomic profiles and histological characteristics, and identified numerous candidate genes involved in sexual development, some well-known and some novel. Significant upregulation of *cyp19a1a*, *foxl2*, *nr5a1a*, *wnt4a*, *hsd3b7*, and *pgr* in females provides strong evidence for the importance of steroidogenesis in ovarian development and maintenance, with *cyp19a1a* playing a central role. *Amh* and *sox8* are two potential key factors that may regulate testicular tissue development in early and late stages, respectively, as they are expressed at higher levels in males than in females, but with slightly different expression timings. Unlike previous descriptions in other fishes, the unique expression pattern of *dmrt1* in *A. clarkii* implied its potential function in both male and female gonads, and we speculated that it might play promoting roles in the early development of both testicular and ovarian tissues.

## 1. Introduction

Sexual reproduction is the most common reproductive mode in multicellular organisms and has been regarded as “the queen of problems in evolutionary biology” [1]. In the evolutionary system of sex-determination mechanisms, teleost fishes exhibit remarkably diverse and complicated sexual developmental patterns, covering almost all types that have been discovered in vertebrates [2]. In contrast to amphibians, reptiles, birds, and mammals, which are mainly gonochoristic with fixed sex after gonadal differentiation, at least 1500 species of teleost fishes display functional hermaphroditism with protogynous, protandrous, and even simultaneous patterns of bisexual gonadal development [3]. Therefore, this unique diversity makes fish an ideal animal model to investigate the formation and evolutionary mechanisms of sex determination and differentiation among the whole vertebrate group.

Clownfish (subfamily Amphiprioninae) are a group of protandrous monogamous teleost fishes that are mainly found in tropical Indo-West Pacific regions living symbiotically with sea anemones [4]. A distinctive characteristic of clownfish is that sexual development is socially controlled and corresponds to changes in body size [5,6]. In general, they live as a small social group composed of a dominant female surrounded by a subdominant male and a variable number of nonbreeders [7]. This fish group exhibits a strong hierarchy of size-based social control that acts as a strict breeding queue [8,9,10]. The dominant individuals control the body size and inhibit the sexual development of low-ranking individuals through aggressive behaviors to achieve their commanding status in the group [11]. Once the dominant female disappears, all subordinates will seize the opportunity to grow and level up in rank. The male partner and the largest nonbreeder will change sex or develop into the dominant female and the subdominant male within a short period of time, respectively [12]. Sometimes, the nonbreeders that have escaped the control of dominators occupy a separate breeding space and could become females without passing through a functional male state [12,13]. In addition, clownfish are small in size, easy to cultivate in captivity, and sexually mature at a young age. Thus, clownfish are particularly suitable for studying the social control of sex determination and differentiation in the laboratory.

The genetic basis is suspected to be one of the key triggers and regulators for creating and maintaining sexual dimorphism and has been extensively studied in species with a genetic sex-determination system. Despite significant research efforts, the master sex-determination genes that drive sex differences have been discovered in only a few species [14,15,16,17,18,19,20,21]. These genes have been reported to be highly divergent and evolve separately in different fish lineages, but the master sex-determination gene in one species usually plays important sex-regulatory roles in others as well. In hermaphroditic species with more flexible sexual fates that respond to environmental stimuli, how genetic cascades interact with environmental factors to orchestrate sexual development has not yet been clarified in any species [22,23]. In particular, the majority of the literature on clownfish describes the gonadal development [24,25], social behavior [26], steroid profiles [27,28], mating strategies [29,30], and the role of single known genes [31,32]. Recently published high-throughput sequencing data of *Amphiprion bicinctus* [33,34] and *A. ocellaris* [35] constitute the only genome-scale resources for this iconic group of species, but they were mainly focused on the genes expressed during the sex change process. The main gene regulatory networks underlying social cues that significantly contribute to sex determination and differentiation are still ambiguous for clownfish.

In this study, we used RNA-sequencing technology to capture the transcriptional profiles of gonad tissues derived from dominant females, subdominant males, and nonbreeders of the yellowtail clownfish *A. clarkii*. This is the first study to quantify and compare gene expression patterns at the genome-wide level among individuals at different social ranks that comprise a social hierarchy. We employed functional annotation and clustering analysis on the differentially expressed genes (DEGs) to identify the main gene regulatory networks that may contribute to the socially-controlled sexual development of *A. clarkii*. Furthermore, the expression localization of two key sex-related genes, *dmrt1* and *foxl2*, were analyzed to confirm their roles in the sexual development process of *A. clarkii*. This work aims to provide insight into the genetic mechanism that underlies socially-controlled sexual development in clownfish.

## 2. Results

### 2.1. Gonadal Histological Characteristics in the Socially-Controlled Groups of A. clarkii

**Figure 1:**
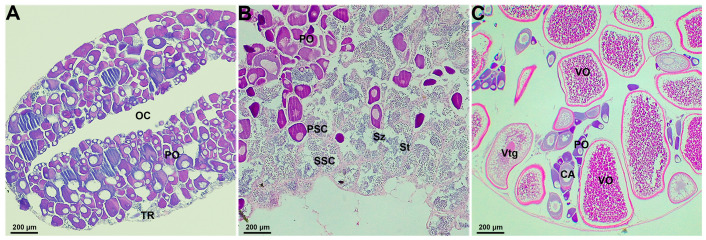
Gonadal histological characteristics of *A. clarkii* at different social ranks. (**A**) Ovotestes of nonbreeders consist of primary oocytes with smaller area of testicular region. (**B**) Ovotestes of males comprise the large area of testicular region in the center surrounded by primary oocytes at the periphery. (**C**) Ovaries of females with all stages of oocytes. TR: testicular region; OC: ovarian cavity; PO: primary oocytes; PSC: primary spermatocyte; SSC: secondary spermatocyte; St: spermatids; Sz: spermatozoa; CA: cortical vesicle stage oocytes; Vtg: vitellogenesis stage oocytes; VO: vitellogenic oocytes. Scale bars = 200 μm.

According to the histological analysis, the gonadal characteristics of each fish in the social group were observed and described. The gonads of nonbreeders were ovotestes that consist mainly of oogonia and primary oocytes with a smaller area of the testicular region in the margins (Figure 1A). As sexual development progressed, the gonads of males showed an increased area of the testicular region that comprised all stages of the spermatogenetic cells (spermatogonia, spermatocytes, spermatids, and spermatozoa) in the center of ovotestes and were surrounded by oogonia and primary oocytes at the periphery (Figure 1B). A small number of scattered primary oocytes were observed embedded in the testicular region (Figure 1B). For females, the gonads were exclusively ovarian tissues that displayed only mixtures of all stages of oocytes without any testicular regions (Figure 1C).

### 2.2. Sequencing, Assembly, and Preliminary Analysis of Gonadal Transcriptomes

To investigate how the social hierarchy affects gonadal development from a genetic perspective, gonadal transcriptome analysis was performed on nonbreeders (NG), males (MG), and females (FG), the main composition of sexual types in a social group. A total of 541.80 million raw reads and 531.29 million clean reads were generated with more than 94% Q30 bases from each library, which suggested that the obtained high-quality sequencing data were reliable and suitable for further analyses (Table 1). It is well known that the analysis results based on the reference genome are more reliable than de novo assembly, and the use of genomic resources from closely related species is common without a genome sequence for the species of interest [36,37]. Among the clean reads, 69.31–74.39% were mapped to the *A. ocellaris* reference genome, which contained 6404 scaffolds with an accumulated length of 880 Mb [38]. The number of mapped reads was sufficient to construct complete transcripts and quantify the expression levels [37,39].

**Figure 2:**
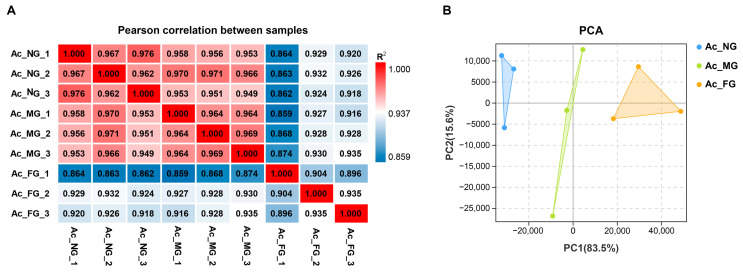
Correlation analysis of samples used in this study. (**A**) Pearson’s correlation coefficients between different samples. “R^2^” represents the square of Pearson’s correlation coefficient. (**B**) Principal component analysis (PCA). NG: nonbreeder gonad; MG: male gonad; FG: female gonad.

In total, 27,495 genes were obtained from the sequencing libraries; among these genes, 24,773 were identified as known genes, accounting for 90.94% of the 27,240 reference genes, and 2722 genes were newly identified. Pearson’s correlation heatmap analysis and PCA were performed with the FPKM of all expressed genes to examine the reproducibility and variability of the biological samples used in this study (Figure 2). The three biological replicates in each group showed small variations with high correlation coefficients (R^2^ > 0.896) that clustered close together, and the data from NG, MG, and FG could be clearly discriminated by PC1, indicating that the data were amenable to further analysis.

### 2.3. Identification and Validation of DEGs

**Figure 3:**
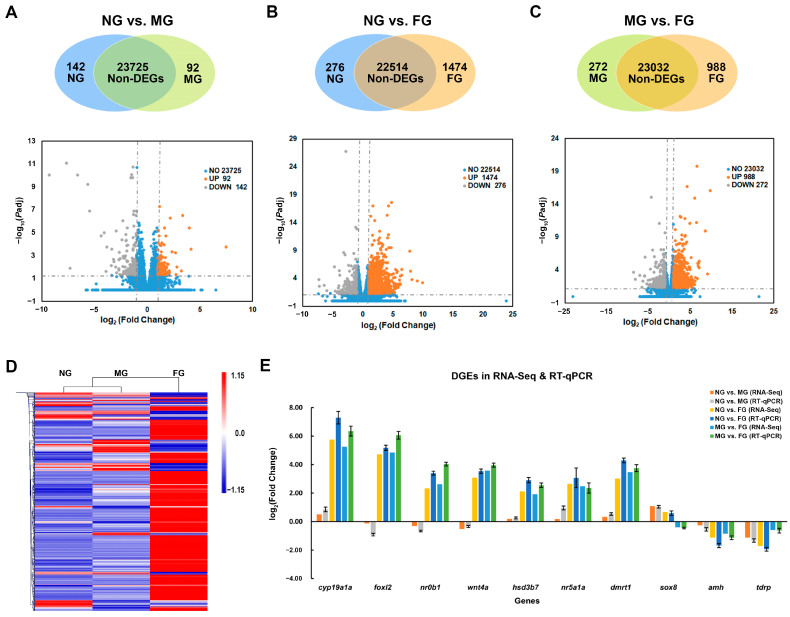
Distribution and validation of DEGs in the comparison groups of NG, MG, and FG. (**A**–**C**) Venn diagrams and volcano plots show the numbers and distribution of DEGs in NG vs. MG, NG vs. FG, and MG vs. FG, respectively. (**D**) Hierarchical clustering analysis of all the DEGs in NG, MG, and FG samples. (**E**) Validation of transcriptomic results by RT-qPCR using 10 selected DEGs.

According to the threshold of |log_2_fold change|≥ 1 and *P*-adj< 0.05, 234, 1750, and 1260 DEGs were identified in the three comparison groups: NG vs. MG, NG vs. FG, and MG vs. FG, respectively (Figure 3A–C). Hierarchical clustering analysis of all DEGs showed that the gene expression patterns of the NG samples displayed higher similarity with those of the MG samples but were distinct from those of the FG samples (Figure 3D), which was consistent with the gonadal developmental profiles.

Ten candidate sex-related DEGs, including *cyp19a1a*, *foxl2*, *nr0b1*, *wnt4a*, *hsd3b7*, *nr5a1a*, *dmrt1*, *sox8*, *amh*, and *tdrp*, were selected for RT-qPCR validation. As shown in Figure 3E, the results showed that the relative expression levels generated from RT-qPCR were similar to those of RNA-Seq, and there was a strong correlation between RT-qPCR and RNA-Seq data (Pearson’s r = 0.99, *p* < 2.2 × 10^−16^), which confirmed the reliability and accuracy of the methods and results presented in this study.

### 2.4. GO and KEGG Functional Enrichment of DEGs

**Figure 4:**
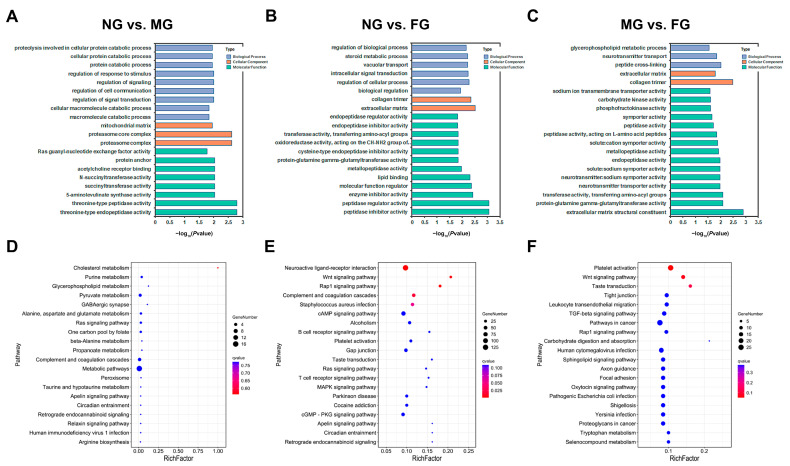
GO and KEGG functional enrichment analysis of DEGs in the comparison groups of NG, MG, and FG. (**A**–**C**) GO profiling of DEGs in all three comparison groups. (**D**–**F**) KEGG pathway analysis of DEGs in all three comparison groups.

GO enrichment analysis revealed that the DEGs in the NG vs. MG group were mainly involved in protein catabolism, stimulus, signal transduction, succinyltransferase activity, 5-aminolevulinate synthase activity and peptidase activity terms (Figure 4A). These DEGs in the NG vs. FG group were mainly involved in steroid metabolic processes (related to ovarian development), vacuolar transport and signal transduction, and had obvious molecular functions in the transport and catabolism of proteins and peptides (Figure 4B). Similarly, a GO term related to the ovarian development glycerophospholipid metabolic process was also enriched in the comparison group of MG vs. FG; in addition, peptidase, symporter, carbohydrate kinase and phosphofructokinase activities might be the major molecular functions of these DEGs (Figure 4C).

KEGG pathway analysis was also performed to categorize the DEGs identified in the three comparisons according to the KEGG database. A total of 68, 197, and 184 KEGG pathways were differentially expressed in the NG vs. MG, NG vs. FG, and MG vs. FG comparisons, respectively. Although only a small portion of the pathways were significantly enriched (*p* < 0.05), the KEGG analysis still provided us with some categorical information of DEGs. In the NG vs. MG comparison, many enriched KEGG pathways were involved in metabolic pathways, such as cholesterol metabolism; purine metabolism; glycerophospholipid metabolism; pyruvate metabolism; alanine, aspartate and glutamate metabolism; one carbon pool; and propanoate metabolism (Figure 4D). In both the NG vs. FG and MG vs. FG comparisons, many enriched KEGG pathways were involved in immunologic processes, such as the Rap1 signaling pathway, complement and coagulation cascades, the B-cell and T-cell signaling pathways, and leukocyte transendothelial migration (Figure 4E,F). In addition, some KEGG pathways involved in sex determination and gonadal development were identified, including the MAPK signaling pathway, Wnt signaling pathway, TGF-beta signaling pathway, and oxytocin signaling pathway.

### 2.5. Screening and Profiling Analysis of Sex-Related Genes

**Figure 5:**
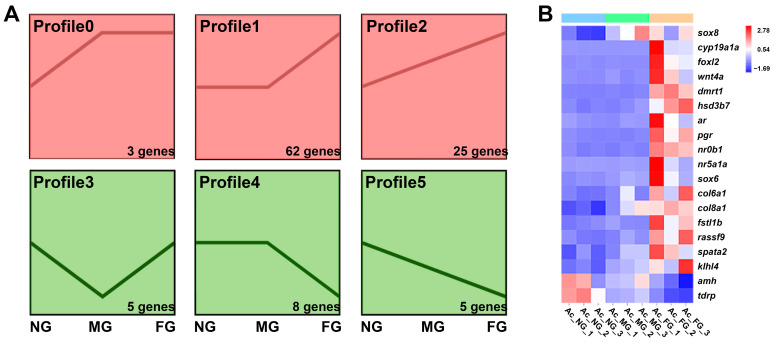
Expression profile analysis of sex-related genes in *A. clarkii*. (**A**) Six expression profiles and the number of sex-related genes in each profile by K-means analysis. (**B**) Heatmap showing the relative expression levels (Z scores) of sex-related genes in NG, MG, and FG.

To identify the genes involved in the sexual regulation of *A. clarkii*, we searched GO terms and KEGG pathways by a set of keywords, including reproduction, gonad, sex, hormone, steroid, oocyte, testis, estrogen, progesterone, and other related signaling pathways. A total of 108 candidate genes were found to be differentially expressed between NG, MG, and FG and then clustered into the following six expression profiles: profile0-profile5 (Table S2 and Figure 5A). The expression of the selected genes that may be important for sexual development [23] of *A. clarkii* are shown in Figure 5B. The expression profiles include these significantly upregulated from NG to MG but leveled off in FG (*sox8*), marked upregulated in FG (*cyp19a1a*, *foxl2*, *wnt4a*, *dmrt1*, *hsd3b7*, *ar*, *pgr*, *nr0b1*, *nr5a1a*), continuously upregulated from NG to MG and then to FG (*sox6*, *col6a1*, *col8a1*, *fstl1b*, *rassf9*, *spata2*, *klhl4*), marked downregulated in FG (*amh*) and continuously downregulated from NG to MG and then to FG (*tdrp*).

### 2.6. Molecular Characterization and Expression Pattern of dmrt1

The full-length cDNA of *Ac*_*dmrt1* was 4046 bp, including a 124 bp 5′-UTR, a 3043 bp 3′-UTR, and an 879 bp ORF encoding a peptide of 292 aa. *Ac*_*dmrt1* possessed a typical conserved DM protein family-specific domain containing six cysteines in the N-terminal region (Figure S1). Molecular phylogenetic analysis showed that Dmrt1 was fairly conserved among vertebrates and that *A. clarkii* was nested deeply inside the teleost clade with *Oreochromis niloticus* and *Symphysodon haraldi* (Figure S2).

**Figure 6:**
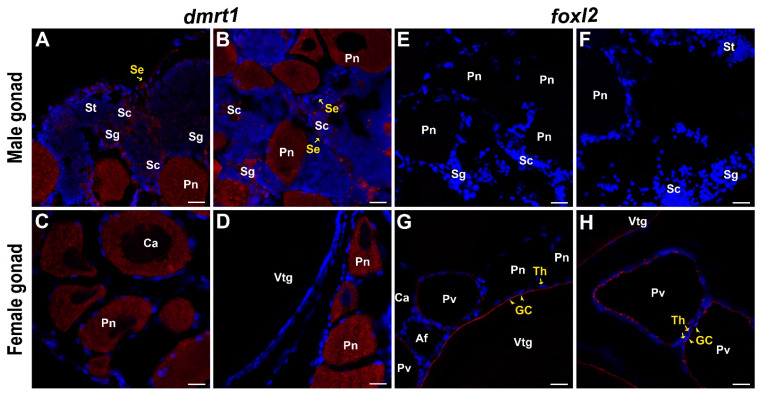
Expression patterns of *dmrt1* and *foxl2* in male and female gonads of *A. clarkii*. (**A**,**B**) The *dmrt1* gene in the male gonad. (**C**,**D**) The *dmrt1* gene in the female gonad. (**E**,**F**) The *foxl2* gene in the male gonad. (**G**,**H**) The *foxl2* gene in the female gonad. Red signal indicates the expression localization of target genes; blue signal indicates cell nucleus; Ca, Cortical Alveoli stage oocytes; Pn, Perinucleolus stage oocytes; Sc, Spermatocytes; Se, Sertoli cells; Sg, Spermatogonia; St, Spermatides; Vtg, Vitellogenic oocytes; Pv, previtellogenic oocytes; Af, atretic follicle; GC, granulosa cells; Th, theca cells; Scale bars = 20 μm.

Signals of *dmrt1* were detected in spermatogonia, spermatocytes, and Sertoli cells in male gonads (Figure 6A). Surprisingly, strong signals of *dmrt1* were also detected in the perinuclear and earlier stages of oocytes in the ovarian region of male gonads (Figure 6B), even continuing to the cortical alveoli stage in female gonads (Figure 6C,D). Even though signals could be detected in both male and female gonads by in situ hybridization, RNA-Seq and RT-qPCR analysis showed that the *dmrt1* level was significantly higher in the female gonads, while the expression level in the male gonads was relatively low (Figure 5B).

### 2.7. Molecular Characterization and Expression Pattern of foxl2

The full-length cDNA of *Ac*_*foxl2* was 2031 bp, including a 240 bp 5′-UTR, an 870 bp 3′-UTR, and a 921 bp ORF encoding a peptide of 306 aa that had a highly conserved Foxlhead protein family-specific domain in the N-terminal region and a histidine-rich region in the C-terminal region (Figure S3). Phylogenetic analysis showed that *A. clarkii* Foxl2 was clustered with the teleost clade and shared the highest similarity with *A. ocellaris*, which confirmed that the sequence we obtained was correct (Figure S4).

In *A. clarkii*, *foxl2* was shown to be a female-specific gene and could only be detected in the granulosa cells of previtellogenic and vitellogenic oocytes in female gonads (Figure 6G,H). No signal was detected in the male gonads (Figure 6E,F). Consistently, RNA-Seq and RT-qPCR analysis revealed that *foxl2* showed a higher expression level in the female gonads (Figure 5B).

## 3. Discussion

Recently, an increasing number of studies have been conducted to analyze the transcript properties of non-model fish species to shed light on the molecular regulatory mechanisms of sexual development. Significant differences in the global transcriptomic profiles of gonads have been detected between males and females, and sex-biased genes have been identified in both hermaphroditic species, such as *Thalassoma bifasciatum* [40] and *Acanthopagrus schlegelii* [41], and gonochoristic species, such as African cichlids [42] and *Cynoglossus semilaevis* [43]. However, almost no previous work has considered how social status affects the molecular networks of sexual development, which in turn regulate individual reproductive behavior, morphology, and physiology. Thus, this study addressed a comparative transcriptomic analysis to examine the relative gene expression across multiple social ranks in the social group of *A. clarkii* rather than just two contrasting sexes, allowing us to explore the molecular sexual regulation mechanism under social control.

### 3.1. Transcriptome Analysis Revealed Similar Gene Expression Profiles between Nonbreeders and Males, Which Supported by Gonadal Histology

Previous studies have shown that these genes, which are predominantly or exclusively expressed in one sex, may reflect the differences in gonadal phenotypes between males and females [44,45]. This sex- and gonad-biased gene expression likely contributes to the phenotypic sexual dimorphism in *Danio rerio* [46] and *A. schlegelii* [41]. In our present work with *A. clarkii*, the differences in mRNA expression levels between females, males, and nonbreeders predicted by transcriptomic data were supported by gonadal histological analysis. Nonbreeders possessed more similar characteristics of gonadal structures and transcript profiles to males, reflecting the consistency of phenotype, genetics, and function, allowing nonbreeders to readily complement male positions. Likewise, primary oocytes in the gonadal ovarian region allow nonbreeders and males to seize the opportunity to quickly switch their sex to females [47]. In the case of gonads for dominant females, only the ovarian tissue is present, while the testis region has completely degenerated, suggesting that the gonad development process of *A. clarkii* is irreversible; that is, females could not revert to males [25,28,48,49].

### 3.2. The Importance of Ovarian Steroidogenesis and the Central Role of Aromatase in the Female Sex Differentiation of Clownfish

In the present study, we examined differences in well-known gene candidates involved in the regulatory network of sexual development. The results showed that a series of genes that participate in the pathway of ovarian steroidogenesis, such as *cyp19a1a*, *foxl2*, *nr5a1a*, *wnt4a*, *hsd3b7*, and *pgr*, presented a strongly upregulated profile in female gonads.

Among these genes, *cyp19a1a* encodes cytochrome p450 aromatase, which is the rate-determining enzyme in estrogen synthesis and converts androgens into estrogens [50,51]. Estrogens produced via the aromatase pathway are considered key steroids for ovarian differentiation in fishes. The expression of *cyp19a1a* has been proven to be increased prior to morphological sex differentiation in gonochoristic fish species [52] and is also involved in the natural sex reversal of hermaphroditic species [53]. Previous studies with *A. clarkii* [32] and *A. bicinctus* [33] showed that *cyp19a1a* is expressed predominantly in female gonads and upregulated in all female categories, similar to the trend found in our study, confirming its essential role in ovarian differentiation and development in clownfish. The *foxl2* could upregulate *cyp19a1a* transcription either directly by binding to the promoter region of *cyp19a1a* or indirectly by interacting with *nr5a1a* to promote ovarian development [54,55]. *Foxl2* is also essential for the maintenance of ovarian granulosa cell identity [56]. In this study, *foxl2* and *nr5a1a* were markedly upregulated in females, displaying a parallel expression profile to *cyp19a1a*, forming a positive feedback loop that has previously been suggested to exist in fish [50], as *foxl2* activates *cyp19a1a*, while estrogens upregulate *foxl2* [54]. In addition, consistent with most previous related studies in fishes, *foxl2* was only localized in the granulosa cells surrounding previtellogenic and vitellogenic oocytes in the female gonads of *A. clarkii* (Figure 6G,H), further confirming the conserved and important role of *foxl2* in ovarian tissue development and maintenance.

In addition, Wnt signaling has already been shown to be another transcriptional regulator of *cyp19a1a*, which could promote ovarian differentiation through the upregulation of gonadal aromatase. Our data showed that a key member of the Wnt signaling cascade, *wnt4a*, was significantly upregulated in females. This gene plays a dose-sensitive role in maintaining germ cell survival in the ovary [57]. Similarly, a steroid synthesis gene (*hsd3b7*) and its receptor (*pgr*) were also found to be markedly upregulated in females, resulting in significant differential expression compared to males and nonbreeders. *Hsd3b7* encodes the key enzyme catalyzing the conversion of pregnenolone to progesterone, and *pgr* encodes the nuclear receptor of progesterone; these proteins have been shown to play a crucial role in regulating reproduction by acting as transcription factors in the somatic cells of female gonads [58]. The above results suggest that ovarian steroidogenesis involving genes such as *cyp19a1a*, *foxl2*, *nr5a1a*, *wnt4a*, *hsd3b7*, and *pgr* is potentially associated with the ovarian development and maintenance of female fate in *A. clarkii*.

### 3.3. Sox8 and Amh Are Potential Key Factors That May Regulate Male Gonad Development in Clownfish

Our analysis revealed two genes known to play clear roles in sex determination, testicular differentiation, and spermatogenesis in teleosts: *sox8* and *amh*, which may be involved in regulating male gonadal development in clownfish. *Sox8* was upregulated in male gonads compared to nonbreeders and then downregulated in female gonads but not significantly, whereas *amh* was upregulated in both males and nonbreeders compared to females in our study.

The *sox8* gene encodes a member of the sry-related HMG box gene family of transcription factors, which is an important determinant for the maintenance of cellular identity in mouse testes [59] and is also an important regulator of adult Sertoli cell function and, thus, male fertility [60]. In teleosts, the role of *sox8* has not been widely considered, but it is known to be more highly expressed in the testis than in the ovary of *Paramisgurnus dabryanus* [61], *Misgurnus anguillicaudatus* [62], and *Paralichthys olivaceus* [63], which suggests that it has an important role in the development and functional maintenance of the testis. The expression profiles of *sox8* in *A. clarkii* were consistent with previous studies, indicating the potential role of *sox8* in male gonad development in protandrous clownfish.

*Amh* (anti-Müllerian hormone) is a multifunctional member of the TGF-*β* superfamily [64], which plays a critical role in regulating gonadal differentiation and function across vertebrates [65]. Our data showed that *amh* expression was sexually differentiated in *A. clarkii*, which is higher in the gonads of males than in females, which is common in numerous fishes, suggesting a potential role in spermatogenesis [66]. *Amh* appears to form an inverse relationship with *cyp19a1a*, as *amh* can directly inhibit *cyp19a1a* expression, which in turn inhibits follicle assembly and growth in females [65]. *Amh* occurs mainly in Sertoli cells surrounding type A undifferentiated spermatogonia of the testis [65,67]. As such, *amh* expression was expected to be elevated along with spermatogonial recruitment during sex development. In this study, we found no significant differences in the expression of *amh* between nonbreeders and males, suggesting that *amh* may be involved in the early development of testicular tissues in *A. clarkii*, while the nonbreeders used in this study were already beyond this developmental stage.

### 3.4. The Special Expression Pattern of dmrt1 Implies Its Potential Function in Oocytes of Clownfish

The *dmrt1* (Doublesex- and mab-3-related transcription Factor 1) has been proven to play a vital role in sex differentiation and testicular development in a variety of vertebrates [68]. In *Oryzias latipes*, the Y chromosome-specific duplication of autosomal *dmrt1* (*dmrt1Y*/*dmy*) was the first sex-determining gene discovered in fish, which was expressed in somatic cells surrounding the germ cells [15,16]. In protandrous *A. schlegeli*, decreased *dmrt1* levels are suspected to be important for initiating a male-to-female sex change because a deficiency of *dmrt1* could result in a loss of germ cells in the testis [69]. In this study, *dmrt1* signals were detected in spermatogonia, spermatocytes, and Sertoli cells in male gonads (Figure 6A,B), similar to many other teleosts, confirming its function in the early development of testicular tissue in *A. clarkii*. However, surprisingly, in perinuclear and earlier stages, oocytes also showed strong signals and even continued into the cortical alveoli stage in female gonads (Figure 6C,D). This higher expression level of *dmrt1* in female gonads was also confirmed by RNA-seq and RT-qPCR. Studies on *D. rerio*, *Gadus morhua* L., *Monopterus albus*, *O. niloticus*, and *Epinephelus akaara* have also shown *dmrt1* signals in developing oocytes; however, the expression level in male gonads is still higher than that in female gonads, or there are multiple isoforms [70,71,72,73,74]. Meanwhile, in *A. bicinctus*, the *dmrt1* was strongly upregulated in males compared to females [33]. The difference in *dmrt1* expression profiles in the two closely related clownfish suggests that there may be slightly different sex-regulation mechanisms. Therefore, we speculated that *dmrt1* might play a promoting role in the early development of oocytes in *A. clarkii*, which differs from other teleosts and requires further functional verification.

### 3.5. The Higher Expression Levels of ar and nr0b1 in Females Imply That Androgens Might Be Closely Related to the Social Status Maintenance of Clownfish

An unexpected finding in our study was that *ar* and *nr0b1* were significantly upregulated in females, in contrast to their higher expression patterns in the male gonads of most other fishes. *Ar* encodes the androgen receptor, which mediates androgen to play significant roles in the differentiation and maintenance of the male reproductive system. Meanwhile, as the immediate precursors for the synthesis of estrogens, androgens can also influence oocyte maturation in females [75]. Additionally, some studies have reported a relationship between androgen concentration and *ar* expression with territorial and aggressive behavioral displays [76,77]. The nuclear receptor dax1 (*nr0b1*) has also been reported to regulate the expression of multiple steroidogenic enzymes in mice [78,79]. The markedly higher expression in females compared to males and nonbreeders of *ar* and *nr0b1*, which contrasts with most teleosts, implies that androgen might be related to the social status maintenance and ovary development of females in clownfish, which calls for a detailed study in the future.

### 3.6. Novel Function of Candidate Genes in the Context of Sexual Development

Several candidate genes with potential roles in the gonadal development process were identified in our study. *Rassf9*, a member of the Ras-association domain family, binds to Ras proteins, which function as the central control element in signal transduction pathways related to cell proliferation, differentiation, and apoptosis [80]. A recent study strongly suggested that the activation of Ras proteins in granulosa cells is crucial for directing normal follicle development [81], which is directly regulated by the progesterone receptor [82]. The genes encoding collagen-alpha-chains (*col6a1* and *col8a1*) function in the growth and development of ovarian follicles and maintenance of proper germline stem cell numbers in both fish and mammals [83,84]. *Sox6* is a transcription factor of the Sox family that may play a role in the sex developmental pathway by regulating spermatogenesis in vertebrates [85]. Follistatin (*fstl*) is expressed at much higher levels during ovarian differentiation in rainbow trout [86,87] and is also known to maintain germ cell survival in the mouse ovary [57]. The *klhl4* gene is a member of the Kelch protein family, which plays an important evolutionarily conserved role in the development of normal oocytes in insects and vertebrates with high levels of expression in the ovary [88]. The transcript levels of *rassf9*, *col6a1*, *col8a1*, *sox6*, *fstl1b*, and *klhl4* were found to be significantly upregulated during sexual development in our study of *A. clarkii*, suggesting that these genes might contribute to gametogenesis in clownfish.

*Tdrp* (testis development-related protein) is a nuclear factor that is predominantly expressed in the spermatogenic cells of the testis and regulates the process of spermatogenesis, especially at the meiotic or postmeiotic stages in mammals [89]. In fish, little is known about the function of *tdrp*. In our study, the role of *tdrp* in the process of sexual development of *A. clarkii* may be completely different from that of mammals, as we found that its expression level was continuously downregulated with the sexual development of males. We speculated that *tdrp* might play a role in maintaining the self-renewal of male germline stem cells; however, this needs to be studied further.

## 4. Materials and Methods

### 4.1. Animals and Sample Collection

The larvae of the *A. clarkii* (standard length: 11.3 ± 0.7 mm) were purchased from a local commercial aquatic farm (Xiamen City, Fujian, China). The fish, three per group, were kept in 100-L tanks with continuously running aerated seawater at 28.0 ± 0.5 °C for twelve months. The fish were fed twice daily with commercial powder or pellet feed. All fish were anesthetized with MS-222 (Sigma Aldrich Chemie GmbH, Germany) to minimize suffering before sampling, followed by histological analysis for gonadal status and social rank. Three fish groups with social hierarchy, consisting of dominant females, subdominant males, and non-breeders, were collected for subsequent analysis.

### 4.2. Gonadal Histological Analysis

The gonads were randomly cut into small pieces and fixed in 4% PFA at 4 °C overnight, dehydrated through an ascending series of graded ethanol concentrations, and then embedded in paraffin blocks. Then, 5-μm thick sections were cut, mounted on slides, and stained with hematoxylin–eosin (HE) for histological examination with a Leica DM500 light microscope (Leica, Wetzlar, Germany) to determine gonadal phases according to the features [12].

### 4.3. cDNA Library Construction and Sequencing

The total RNA of the gonadal tissues was isolated using RNAiso Plus (Takara, Shiga, Japan) method. The quality and quantity of extracted RNA were evaluated using NanoPhotometer® spectrophotometer (IMPLEN, Calabasas, CA, USA), Bioanalyzer 2100 system (Agilent Technologies, Carpinteria, CA, USA), and Qubit^®^ 2.0 Fluorometer (Life Technologies, Carlsbad, CA, USA). The RNA samples that satisfied RIN ≥ 6.8 and 1.8 < OD260/280 < 2.2 were used for further cDNA library construction. Sequencing libraries were established using NEBNext^®^Ultra™ RNA Library Prep Kit for Illumina^®^ (New England Biolabs, Ipswich, MA, USA) by enrichment of mRNA using poly-T oligo-attached magnetic beads. Double-stranded cDNA was synthesized using random hexamer primer and purified with the AMPure XP system (Beckman Coulter, Brea, CA, USA). The cDNA libraries were obtained by PCR enrichment, and then paired-end sequencing was conducted using the Illumina HiSeq platform (Novo Gene Biological InfoTech Ltd., Tianjin, China) to obtain 125 bp/150 bp reads. All sequencing datasets can be found in the NCBI Sequence Read Archive (SRA) database under accession number PRJNA850025.

### 4.4. Quality Control and Transcriptome Assembly

The raw reads were filtered using in-house perl scripts to remove the low-quality (quality scores ≤ 5) and adapter/poly-N-contaminated reads. At the same time, Q20, Q30, and GC content of clean reads were calculated. Because clownfishes are a very homogeneous group [90], and the genomic resources from a closely related species would be helpful for improving transcriptome assembly [91], the paired-end clean reads were aligned to the high-quality *A. ocellaris* reference genome (NCBI: PRJNA407816) using Hisat2 v2.0.5 [92].

### 4.5. Identification of DEGs

FeatureCounts v1.5.0-p3 [93] was used to count the number of reads mapped to each gene. FPKM was calculated based on the length and reads count to estimate the gene expression level. Pearson correlation matrix and principal component analysis (PCA) were performed using the Pvclust and PCAtools in the R package to assess the reproducibility and variability of biological samples based on the overall gene expression levels. Differential expression analysis between two groups was performed using the DESeq2 R package [94]. Genes with a minimal 2-fold difference in expression level (|log2fold change| ≥ 1) and *P*-adj < 0.05 were identified as DEGs. Gene Ontology (GO) enrichment and KEGG (http://www.genome.jp/kegg/, accessed on 1 April 2022) pathway analyses were implemented by the clusterProfiler R package to predict the functions of DEGs. Corrected *p*-value < 0.05 were considered significantly enriched. K-mean clustering was performed using the k-mean function in the R package to group DEGs with similar expression patterns in entire groups. The genes clustered into the same cluster were considered to have similar expression patterns.

### 4.6. Quantitative Real-Time PCR (RT-qPCR) Verification

Ten genes that showed significantly different expression levels in at least one comparison group were selected to perform RT-qPCR analysis. RNA samples used for RT-qPCR were the same used for RNAseq. First-strand cDNA was synthesized using PrimeScript™ RT reagent kit with gDNA Eraser (Takara, Shiga, Japan), and RT-qPCR was carried out with 7500 Fast Real-time PCR System (Applied Biosystems, Foster, CA, USA) using TB Green Premix DimerEraser (Takara, Shiga, Japan). Specific primers were designed using Primer Premier 5.0, as listed in Table S1, and the amplification efficiency was verified by a cDNA dilution series. The 2^−ΔΔCt^ method was used to calculate relative gene expression with *β-actin* as an internal reference gene [95]. All of the samples were analyzed in triplicate, and the data were shown as mean ± standard deviation (SD). One-way analysis of variance (ANOVA) followed by post-hoc Duncan’s multiple range tests was employed to determine the difference between groups using SPSS Statistics 26.0 (SPSS, Chicago, IL, USA).

### 4.7. Sex-Related Gene Sequence Confirmation and In Situ Hybridization (ISH) Analysis

Full-length cDNA sequences of *dmrt1* and *foxl2* genes were obtained by the rapid amplification of the cDNA ends (RACE) method (primers listed in Table S1). Multiple alignment and homology of teleost amino acid sequences were carried out using ClustalX software (http://www.clustal.org/clustal2/, accessed on 16 May 2022). Phylogenetic analysis of each gene was performed with MEGA 7.0 by the Neighbor-Joining method (Boost-strap = 1000).

For in situ hybridization, sense and antisense RNA probes were synthesized by PCR (primers listed in Table S1) using the digoxigenin (DIG) RNA labeling kit (Roche Applied Science, Mannheim, Germany) and purified using the NucleoSpin^®^ RNA Clean-up Kit (Macherey-Nagel, Düren, Germany). Before the in situ hybridization, the sections were de-waxed, rehydrated, permeabilized, refixed, acetylated, and prehybridized with the hybridization buffer for 2 h at 65 °C. Hybridization with RNA probes was carried out at 65 °C overnight in the dark. The hybridization signals were detected using anti-digoxigenin-AP Fab fragments (Roche Applied Science, Mannheim, Germany) and HNPP Fluorescent Detection Sets (Roche Applied Science, Mannheim, Germany). The slides were stained with Hoechst solution, mounted, and observed under Zeiss LSM 780 confocal fluorescence microscope (Zeiss, Jena, Germany).

## 5. Conclusions

In summary, our study provided valuable gonadal gene expression data of nonbreeders, males, and females in the *A. clarkii*. We compared and identified numerous DEGs in the three comparison groups. The global gene expression patterns from the NG samples displayed higher similarity with those from the MG samples but were distinct from those of the FG samples, which was consistent with the gonadal histological characteristics reflecting the consistency of phenotype, genetics, and function. The distinct upregulation of *cyp19a1a*, *foxl2*, *nr5a1a*, *wnt4a*, *hsd3b7*, and *pgr* in females provided strong evidence that the important role of steroids in ovarian development and maintenance, with the *cyp19a1a* gene playing a central role. *Amh* and *sox8* were two potentially key factors that may regulate early and late testicular tissue development, respectively, as they both showed higher expression levels in males than in females but with slightly different expression timings. Novel genes such as *rassf9*, *col6a1*, *col8a1*, *sox6*, *fstl1b*, *klhl4*, and *tdrp* that are potentially involved in the sexual development of *A. clarkii* were identified. Moreover, the expression localization analyses of key sex-related genes showed that the expression patterns of *foxl2* were similar to those of gonochoristic and other hermaphroditism fishes, while the unique expression pattern of *dmrt1* implied its potential function in both male and female gonads of clownfish. The results of this study provide insights into the potential molecular mechanisms that regulate the sexual development of socially-controlled hermaphroditic fish and further extend our knowledge about the maintenance and sexual plasticity of vertebrates.

## Figures and Tables

**Table 1:** Sequencing and assembly statistical for the gonadal transcriptomes of *A. clarkii*.

Samples	Raw Reads (×10^6^)	Clean Reads (×10^6^)	Q20 (%)	Q30 (%)	GC (%)	Total Map (%)	Exon (%)	Intron (%)	Intergenic (%)
Ac_NG_1	68.15	66.85	98.44	95.48	50.01	70.77	73.49	4.71	21.81
Ac_NG_2	69.56	68.47	98.32	95.16	50.09	70.67	72.42	4.73	22.86
Ac_NG_3	61.47	60.27	98.16	94.83	49.92	69.31	71.76	4.97	23.27
Ac_MG_1	55.75	54.81	98.28	95.03	50.51	71.00	72.86	4.83	22.31
Ac_MG_2	59.84	58.46	98.14	94.75	49.94	71.26	75.04	4.41	20.56
Ac_MG_3	60.32	59.24	98.24	94.99	49.90	71.88	75.62	4.36	20.02
Ac_FG_1	61.39	59.89	98.32	95.16	49.62	74.39	79.70	3.97	16.33
Ac_FG_2	53.66	52.60	98.14	94.84	49.89	70.00	72.11	4.65	23.25
Ac_FG_3	51.66	50.70	98.26	95.00	49.11	72.57	78.28	4.25	17.47
Total	541.80	531.29	/	/	/	/	/	/	/

## Data Availability

All sequencing datasets can be found in the NCBI Sequence Read Archive (SRA) database under accession number PRJNA850025.

## Supplementary Materials

The supporting information can be downloaded at: https://www.mdpi.com/article/10.3390/ijms23169085/s1.
