# Supplementary material for: Transcriptome Profiling and Expression Localization of Key Sex-Related Genes in a Socially-Controlled Hermaphroditic Clownfish, Amphiprion clarkii
Source: Int J Mol Sci. 2022 Aug 13;23(16):9085. doi: 10.3390/ijms23169085 (PMC9409170; doi:10.3390/ijms23169085)
Supplement: Supplementary file 1 [file ijms-23-09085-s001.zip › Supplementary figures.pdf]

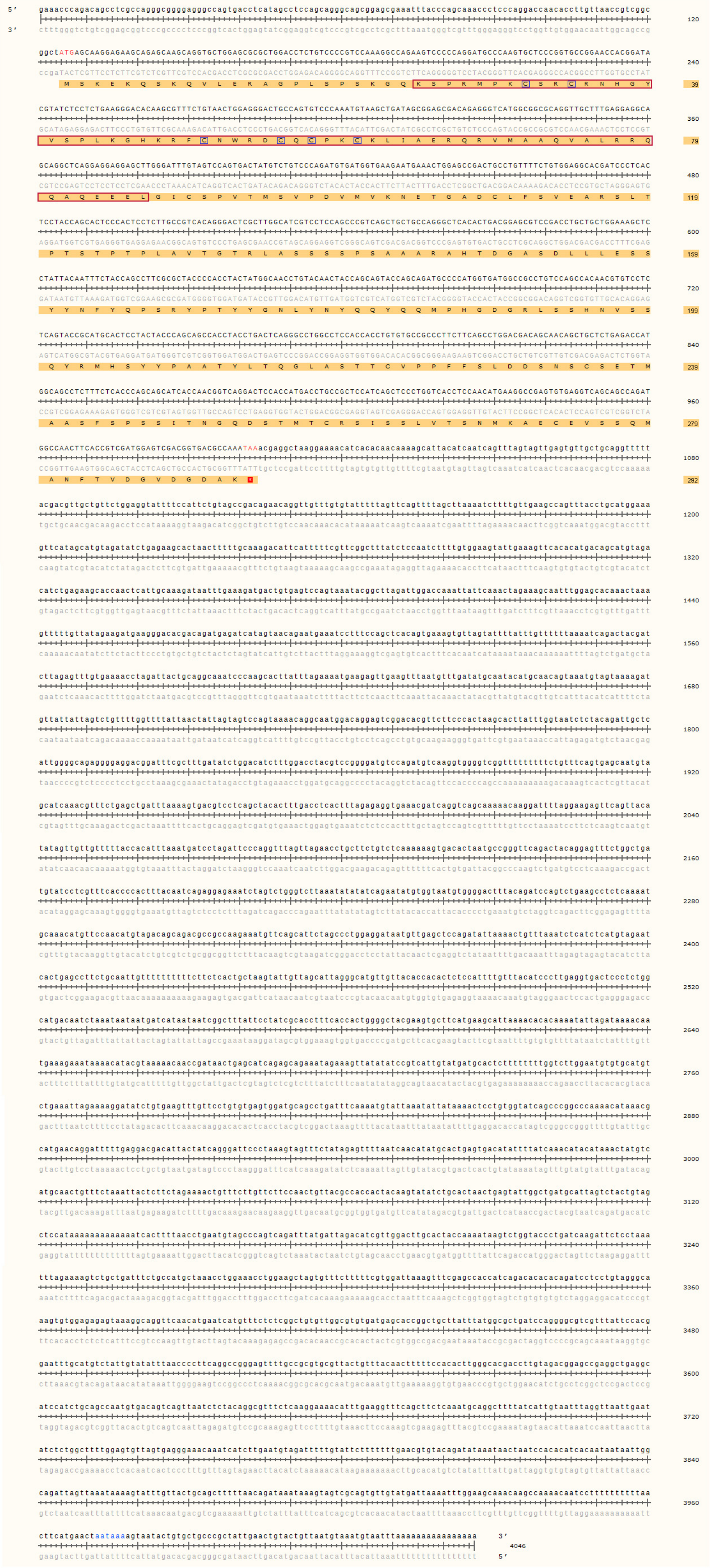

Figure S1. The mRNA and amino acid sequences of *Ac_dmrt1*. The DM domain is boxed in red.

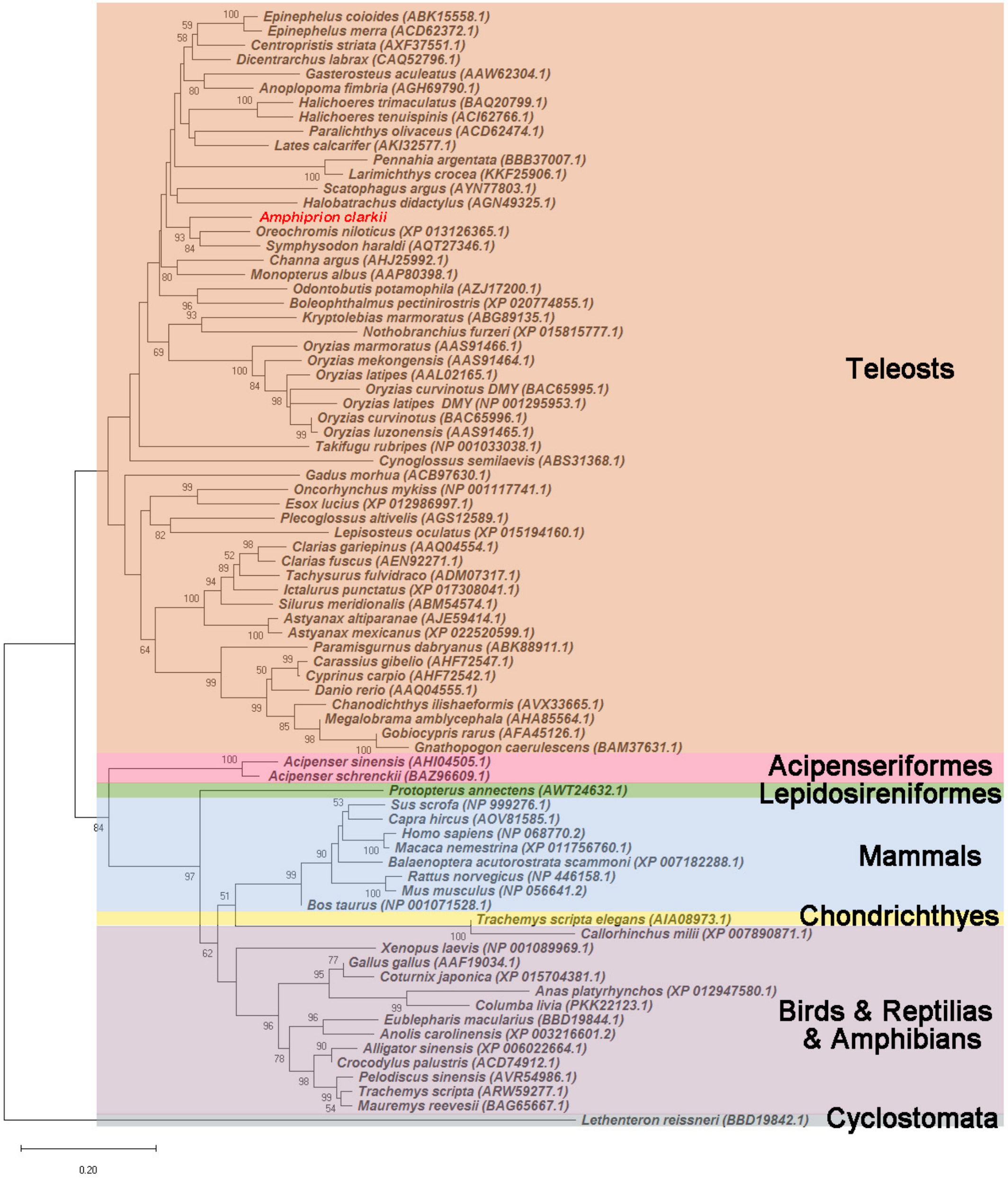

Figure S2. Phylogenetic tree based on Dmrt1 amino acid sequences.

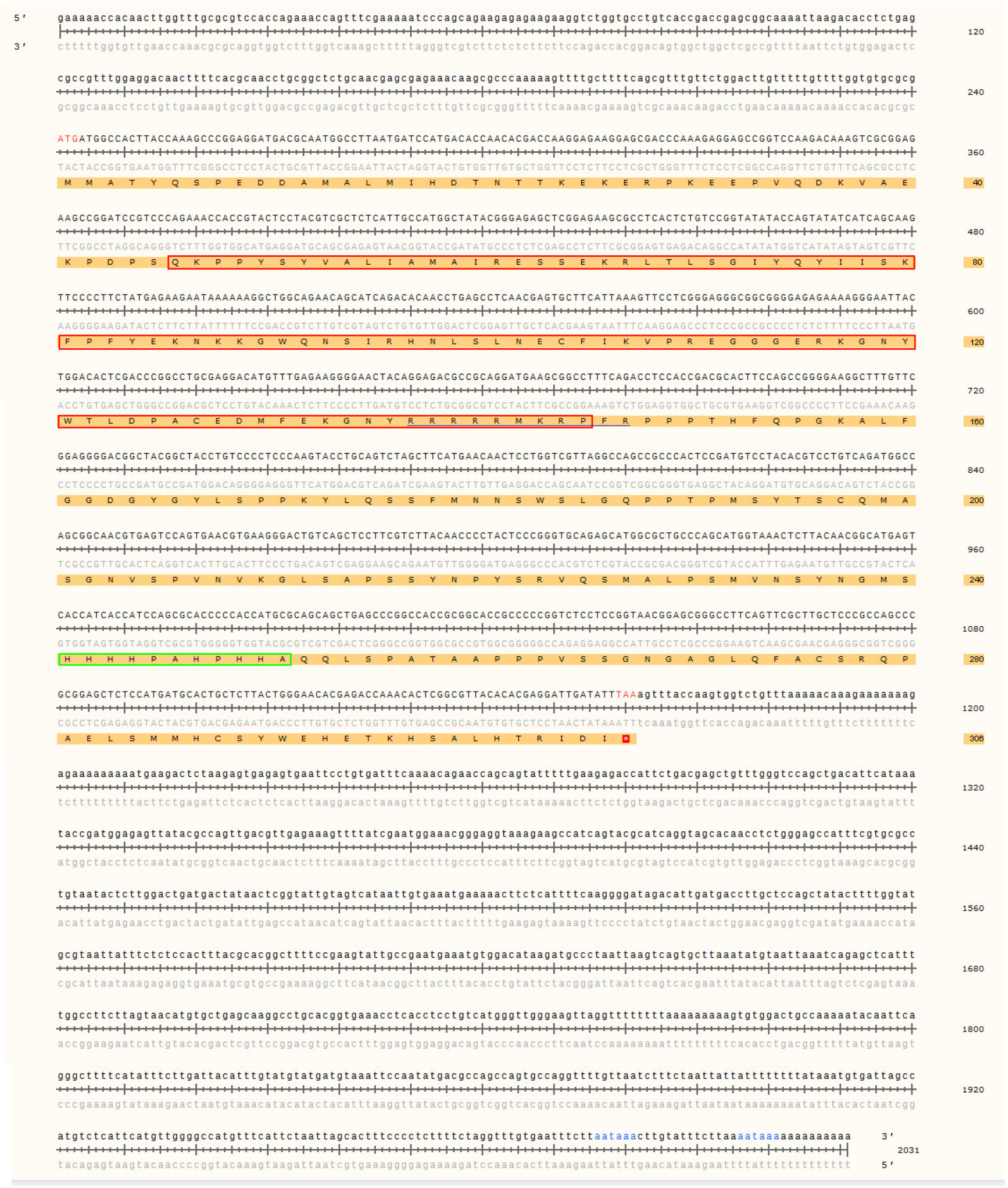

Figure S3. The mRNA and amino acid sequences of *Ac_foxl2*. The forkhead domain is boxed in red.

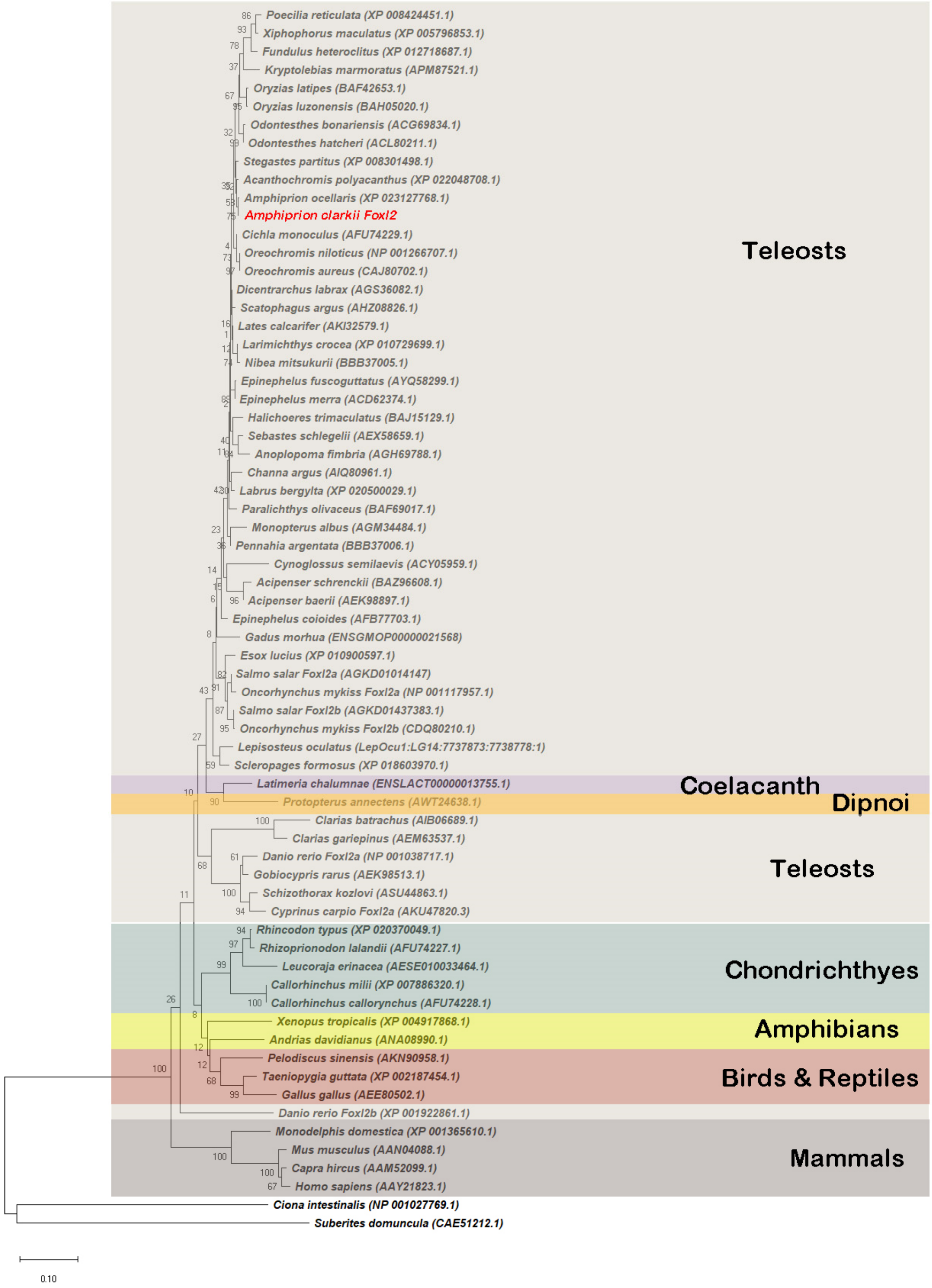

Figure S4. Phylogenetic tree based on Foxl2 amino acid sequences.
